# Protective effects of omega-3 fatty acids in dogs with myxomatous mitral valve disease stages B2 and C

**DOI:** 10.1371/journal.pone.0254887

**Published:** 2021-07-15

**Authors:** Priscilla Regina Nasciutti, Aline Tavares Moraes, Thaiz Krawczyk Santos, Karine Kelly Gonçalves Queiroz, Ana Paula Araújo Costa, Andressa Rodrigues Amaral, Rodrigo Fernando Gomes Olivindo, Cristiana Ferreira Fonseca Pontieri, Juliana Toloi Jeremias, Thiago Henrique Annibale Vendramini, Marcio Antonio Brunetto, Rosângela de Oliveira Alves Carvalho

**Affiliations:** 1 Veterinary Cardiology Service of the Veterinary Hospital of the School of Veterinary Medicine and Animal Science, Federal University of Goias, Goiania, Brazil; 2 Nutrition and Production Department, Pet Nutrology Research Center, School of Veterinary Medicine and Animal Science, University of Sao Paulo (USP), Pirassununga, Brazil; 3 Nutritional Development Center, Grandfood Indústria e Comércio Ltda (Premier Pet), Dourado, Brazil; Scuola Superiore Sant’Anna, ITALY

## Abstract

Myxomatous mitral valve disease (MMVD) is characterized by thickening of the valve leaflets and omega-3 (ω-3) supplementation has been associated with modulation of blood pressure (BP) and heart rate, improvement of doppler echocardiographic indices, antiarrhythmic, anti-inflammatory and anti-dislipidemic effects in dogs and humans, although prospective studies of it single use are still absent in the veterinary literature. The objective of this study was to evaluate the influence of ω-3 supplementation in dogs with MMVD. Twenty-nine dogs were followed quarterly for 12 months by clinical evaluation, arterial blood pressure, electrocardiography, doppler echocardiography, thoracic radiography and laboratory tests including inflammatory mediators and cardiac biomarker blood concentrations. The dogs were classified in stages B2 and C, according to the classification proposed by ACVIM 2019. They were randomly assigned to either ω-3 group (ω-3G) or control group (CG). The ingestion of ω-3 reduced the chance of developing arrhythmias by 2.96 times (p = 0.003). The vertebral heart size (VHS) measurements were higher in the control group (p = 0.033). In conclusion, at the dosages used in this study, ω-3 dietary supplementation reduces the volumetric overload, has antiarrhythmic effect and keeps dogs with B2 and C stages of MMVD in milder stages of the disease.

## 1. Introduction

Myxomatous mitral valve disease (MMVD) represents 75% of cardiovascular diseases in canine and is recognized as the leading cause of cardio insufficiency and main cause of death in dogs older than eight years of age [1,2]. MMVD mainly affects the left atrioventricular valve progressively, whose changes can be initially recognized by the presence of cardiac murmur on clinical examination and diagnosed by echocardiography when observing valve regurgitation resulting from degeneration of the valve leaflets and reduction of its functional capacity [3].

The repercussions of valvular functional disability make ventricular emptying difficult, causing reflux/regurgitation in the atrial direction that changes the cardiac output (CO), heart rate (HR) and vascular hemodynamic. Such changes lead to left ventricular hypertrophy to overcome the resulting peripheral vascular resistance. This myocardial remodeling is accompanied by an inflammatory response with cytokine release (TNF-α and IL-1) that perpetuates the valve degeneration process and favors the consumption of muscles for energy supply, a process called cachexia [3–5].

Cachexia is a complex and multifactorial metabolic syndrome that leads to anorexia, increased energy requirements (up to 30%) and depletion of energy storage, especially muscles. In addition, it is related to a significant decrease in life expectancy [6,7]. According to INUI et al. [8], 22% of deaths may be caused by this condition and, additionally to the progressive heart failure contribute to the poor prognosis of the disease.

The use of omega-3 (ω-3) polyunsaturated fatty acids for cardioprotective effect was initially questioned in 1970s when population data detected low occurrence of myocardial infarction and coronary artery disease in Greenland eskimos fed on large amounts of cold-water fish [9–11]. Since then, several human studies have already pointed out the antilipidemic, antiarrhythmic, antinflammatory and antihypertensive effects of eicosapentaenoic (EPA) and docosahexaenoic (DHA) fatty acids [9,12–15], which are the main biologically active product of omega-3 after its conversion. After 30 years, in a literature review based on human medicine, the protective effects against sudden death caused by cardiac events were acknowledged by their ability to control atrial and ventricular arrhythmias, reduce heart rate (HR) and increase their variability [15].

Similarly, research in veterinary medicine has been developed with the same purpose, so that the dietary inclusion of EPA and DHA has already been shown to be beneficial in dogs with arrhythmogenic right ventricular cardiomyopathy because it reduces the inflammatory status and number of premature ventricular complexes; as well as in dogs with dilated cardiomyopathy by reducing interleukin 1 (IL-1) concentrations and improving cachexia [16–19].

Given all these beneficial effects, ω-3 has been recognized as a cardioprotective nutraceutical in human and canine patients. However, prospective clinical studies evaluating the effects of single ω-3 dietary supplementation in dogs with B2 and C stages of MMVD have not yet been performed.

## 2. Materials and methods

All the methodology procedures were in accordance to the Brazilian Society of Science in Laboratory Animals recommendations and was previously submitted for analysis and approval by the Animal Use Ethics Committee of the Federal University of Goias (UFG), protocol 094/14.

### 2.1 Animals and experimental design

The experimental study was performed at the Cardiology Laboratory of the Veterinary Hospital of the Veterinary and Animal Science School (EVZ) of UFG and at the Specialized Laboratory for Scientific Analysis (LEAC) in São Paulo, both in Brazil.

Twenty-nine male and female dogs owners, who were attended in the Cardiology sector of the Veterinary Cardiology Service of the Veterinary Hospital of the School of Veterinary Medicine and Animal Science, Federal University of Goias and classified of stage B2 or C were enrolled in this study and treated according to Keene et al. [2] consensus.

All animals were double-blinded randomized in two experimental groups: ω-3 group (ω-3G) or Control group (CG). The nutrient composition of the diets is described in Table 1.

**Table 1:** Nutrient composition and ingredients of the experimental diets.

Nutrient	Control and omega-3 supplemented diet*	
	% original matter	g/100 kcal
Moisture	6.9	2.5
Crude protein	25.2	6.0
Fat (acid hydrolysis)	19.6	4.5
Crude fiber	1.7	0.8
Ash	4.2	1.5
EPA*	0.4	0.1
DHA*	0.3	0.07
Na	0.15	0.04
ME (kcal/g)	4.2	
Ingredient list	Rice grits, chicken bowels flour, chicken fat, corn gluten meal 60%, isolated pork protein, beat pulp, fish oil, whole egg powder, pork fat, defatted rice bran, dry brewer’s yeast, hydrolyzed pork liver, potassium citrate, calcium carbonate, potassium chloride, soy lecithin, taurine, yeast cell wall (MOS source), vitamin and mineral premix, antifungal, absorbents of mycotoxins, methionine and antioxidant.	

*Described amounts only included in the supplemented diet. Kcal: Kilocalories; EPA: Eicosapentaenoic acid; DHA: Docosahexaenoic acid; Na = sodium; ME = metabolizable energy.

ω-3 dose for supplementation was based on Freeman’s [20] recommendation in order to obtain, approximately, 150mg EPA+DHA/100kcal (final 170mg in order to consider possible losses) and a 1.5 EPA/DHA ratio (final 1.3).

Food intake was calculated in order to achieve their daily energy requirement (DER) according to NRC [21] recommendations through the equation:

$$DER\;(\frac{kcal}{day})=95\times {\mathrm{body}\;\mathrm{weight}}^{0.75}$$

The animals were prospectively followed every three months for 12 months (T0, T3, T6, T9 and T12). Clinical assessments of vital parameters, routine serum hematological and biochemical laboratory tests including the concentration of inflammatory markers: interleukin-6 (IL-6), interleukin-1β (IL-1β), tumor necrosis factor alpha (TNF-α), C-reactive protein (CRP) were performed at each experimental time. NT-proBNP marker was evaluated at T0, T3, T6 and T9.

Body condition score (BCS) and muscle mass score (MMS) was assessed at all meetings according to Laflamme (1997) [22] and Baldwin et al. (2010) [23], respectively by the same person (P.R.N.).

The animals were auscultated in mitral, aortic, tricuspid and pulmonary focus in order to identify and classify the cardiac murmur degrees in a scale of one to six according to Camacho and Mucha (2014) [24].

Finally, as for cardiovascular evaluation, a non-invasive arterial blood pressure (ABP), electrocardiography (ECG), echodopplercardiography (ECO) and thoracic radiography was performed.

### 2.2 Laboratory analysis

Fasting blood samples were obtained by aseptically jugular venepuncture for complete blood count in ABC 7 Vet Animal Blood Counter haematological analyzer (Gurnee, IL, USA) and serum profile check-up in Wiener Lab CM 250 model analyzer (Rosario, Santa Fe, Argentina) and Roche Cobas b 121 model Blood Gas analyzer (Basel, Switzerland) for concentration of potassium, sodium and chloride electrolytes.

Inflammatory cytokines were analyzed by enzyme-linked immunosorbent assay (ELISA) in LEAC—São Paulo in Stat Fax Model 2100 (Awareness Technology), reader in MultiCalc program. For IL-6 and TNF-α analysis the Canine Cytokine Magnetic Bead Panel Multiplex Canine Kit CYTOMAG-90K-02 (Millipore, St Charles, Missouri—United States) was used. PCR dosages were performed using the canine ECA0006-PCR kit (Wuhan Fine Biological Technologic Co—China). Dosages of IL-1β were assessed using the canine E-ELC-IL1B kit (Ray Biotec/Norcross—United States).

Analysis of NT-proBNP was conducted at IDEXX laboratory with immune assay kit Canine CardiopetTM proBNP (IDEXX Laboratories, Maine, EUA).

### 2.3 Cardiovascular evaluation

ABP was measured using the non-invasive vascular doppler method with Parks Medical Ultrasonic Doppler 812® (Parks Medical, USA) and Henik Gamma G5® sphygmomanometer (Heine, Germany) [25–27].

In each evaluation, five consecutive systolic blood pressure (SBP) measurements were obtained, discarding the highest and lowest measurements, and considering the arithmetic mean of the three remaining measurements as the final value.

In order to obtain the electrocardiogram (ECG) records, computer ECG acquisition module/ECG-PC, version 6.2—revision 1 Copyright © 1997–2011—Brazilian Electronic Technology–TEB was used at 50mm/s. All bipolar, unipolar and precordial derivations were obtained simultaneously for five minutes. The interpretations of the tracings were performed in the DII derivation, according to Petrie [28].

The 24-hour ambulatory electrocardiography (Holter) was performed with the Cardiolight® ECG Holter device (Cardios—São Paulo, Brazil) in two precordial derivations (rV2 and V4). Three electrocardiographic channels were recorded from these modified thoracic derivations. The information was recorded on the device’s memory card and later transferred to the reading software for data interpretation.

The Doppler echocardiographic study was performed using the Doppler echocardiography device (My Lab30Vet—Esaote/Pie Medical) and a 5.0/7.5MHz multifrequency transducer. Measurements of the cardiac structures obtained at the level of papillary muscles were performed in M mode by bidimensional orientation.

The qualitative evaluation of the heart was obtained by the bidimensional mode (2D), in order to obtain the left atrium (LA) to aorta (Ao) ratio (La/Ao) and orientation for the M-mode images, through which quantitative analysis in systole and diastole of the cardiac chambers, left ventricular free wall thickness (LVFW) and the interventricular septum (IVS) measurements was performed and evaluation of valvular leaflets, thus allowing calculations of functional parameters such as shortening fraction (SF) and ejection fraction (EF) [29].

Stroke volume (SV) and CO (cardiac output) indices were measured using aortic flow and its diameter. Pulsed Doppler was also used to measure diastolic function by obtaining the E-wave, A-wave velocities and isovolumetric relaxation time (IVRT). Doppler echocardiograms were performed and analyzed according to the criteria of the American Society of Echocardiography and the Echocardiography Committee of the American College of Veterinary Internal Medicine [29].

Thoracic X-ray examinations were performed using a Philips KL.74/20.40 fixed x-ray machine (Philips Healthcare®, Biassono, Italy) with a capacity of 500mA. Cardiac silhouette analysis was performed using the Vertebral Heart Size (VHS) measurement according to Buchanan and Bucheler [30].

### 2.4 Calculations and statistical analysis

For the analysis of qualitative variables, Fisher’s exact test was used, since there were categories with zero animals, and Pearson’s chi-square test. Results were presented as a percentage. For quantitative variables, the Mann-Whitney test was used for comparison of the treatment and the Kruskal-Wallis test for comparison within moments within the same group, since the data was not normally distributed. Results were presented as means and standard deviation. A significance level of 0.05 was set. The survival curve graph was constructed using the Kaplan Mayer procedure and, in order to compare survival curves, the Log Rank method was used. The Cox regression model was used to identify the relationship between survival time and treatment. To assess whether there was correlation between the dosages of inflammatory markers with the BCS and MMS, the Spearman correlation test was performed.

## 3. Results

Twenty-nine male (n = 17) and female (n = 12) dogs with B2 or C MMVD were selected. Breeds included were Poodle (n = 12), Pinscher (n = 6), Lhasa Apso (n = 2), Teckel (n = 2), Maltese (n = 1), Yorkshire Terrier (n = 1) and crossbreed (n = 5). Dogs had a mean weight of 6.10 ± 2.68 kg and mean body condition score (BCS) of 5.78 ± 2.69 according to the nine-point scale of Laflamme (1997) [22].

The ω-3G composed of 16 dogs with an average age, weight, BCS and MMS of 11.56 ± 2.76 years, 5.94 ± 2.49 kg, 5.69 ± 2.22, 2.75 ± 0.45. Among those, 9 classified in stage B2 and 7 in stage C and received dry food for cardiac dogs supplemented with ω-3 in the average doses of 54.2mg/kg EPA and 40.65mg/kg DHA. CG was composed of 13 dogs with an average age, weight, BCS and MMS of 11.67 ± 2.02 years, 6.55 ± 2.81 kg, 5.46 ± 1.33 and 2.85 ± 0.38, respectively. Among those, 7 were classified in stage B2 and 6 in stage C, which received the same dry food of ω-3G, although without EPA and DHA inclusion.

Initially the groups were similar when clinical signs such as cough, syncope and seizures were present. Except for the presence of cyanosis, which occurred from T0 more frequently in the control group. No differences were found for clinical parameters, for BCS and MMS between the experimental groups at the beginning of the study (T0) (Table 2).

**Table 2:** Averages of clinical baseline parameters and evolution of heart rate and respiratory rate of dogs with stage B2 and C myxomatous mitral valve disease with omega-3 dietary supplementation or not for 12 months.

		Control		Omega-3			
Variables	Times	n	Averages	n	Averages	p*	p**
**HR (bpm)**	T0	13	149.00 ± 28.00	16	154.00 ± 45.00	0.363	0.983
	T3	10	144.00 ± 23.00	10	146.00 ± 40.00	0.853	
	T6	9	139.00 ± 27.00	8	138.00 ± 29.00	0.673	
	T9	9	137.00 ± 14.00	8	144.00 ± 32.00	0.606	
	T12	7	140.00 ± 28.00	6	148.00 ± 30.00	0.755	
	p***		0.971		0.711		
**RR (mpm)**	T0	13	109.00 ± 63.00	16	93.00 ± 55.00	0.525	0.370
	T3	10	64.00 ± 49.00	10	108.00 ± 56.00	0.853	
	T6	9	64.00 ± 40.00	8	84.00 ± 56.00	0.370	
	T9	9	70.00 ± 58.00	8	85.00 ± 61.00	0.167	
	T12	7	69.00 ± 50.00	6	111.00 ± 43.00	0.530	
	p***		0.176		0.676		

HR: Heart rate; bpm: Beats per minute; RR: Respiratory rate; mpm: Movements per minute; p*: Difference between treatments; p**: Difference between times; p***: Difference in treatment by time interaction Kruskal-Wallis test at 5% of significance.

**Fig 1:**
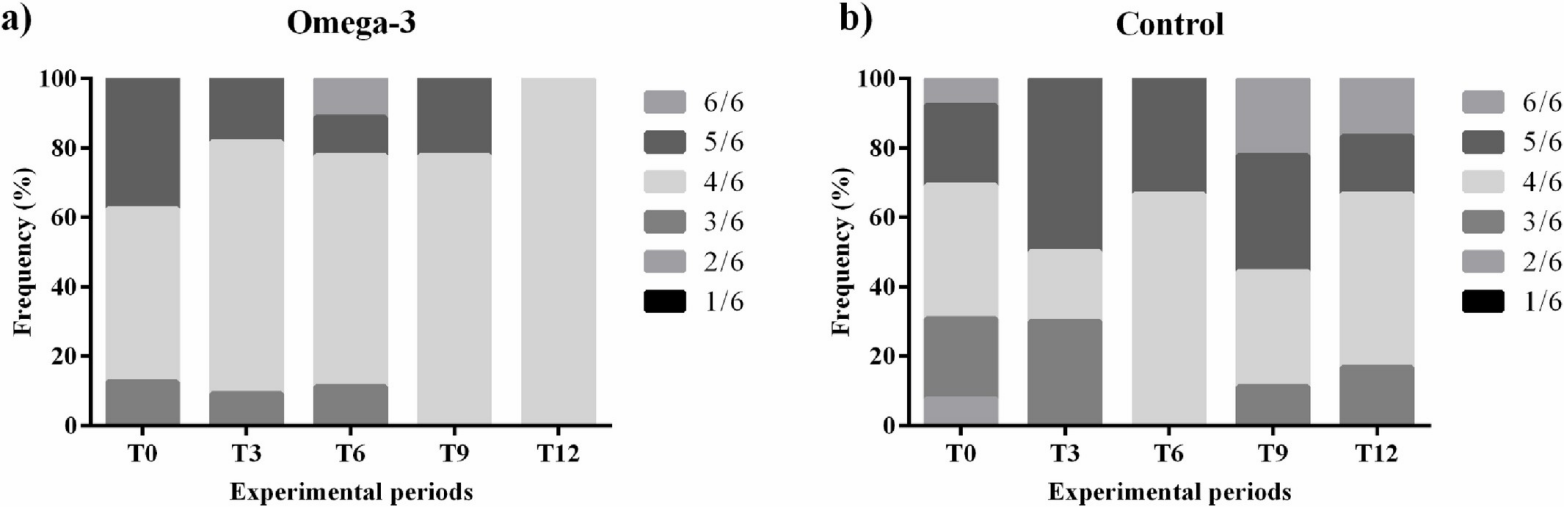
Mitral valve murmur grading frequency of dogs with stage B2 and C myxomatous mitral valve disease supplemented with omega-3 (a) or not (b).

During cardiac auscultation, rhythmic and normophonetic sounds were found with the presence of systolic murmur in all dogs, except for one dog from CG, which started arrhythmic at T6. Considering the evolution of mitral murmur, a lower evolution was observed in patients in the ω-3G group than in the CG. Only patients in the CG presented heart thrill (murmurs grade 5/6 and 6/6) at the last evaluation. Murmur grading frequencies are illustrated in Fig 1.

**Fig 2:**
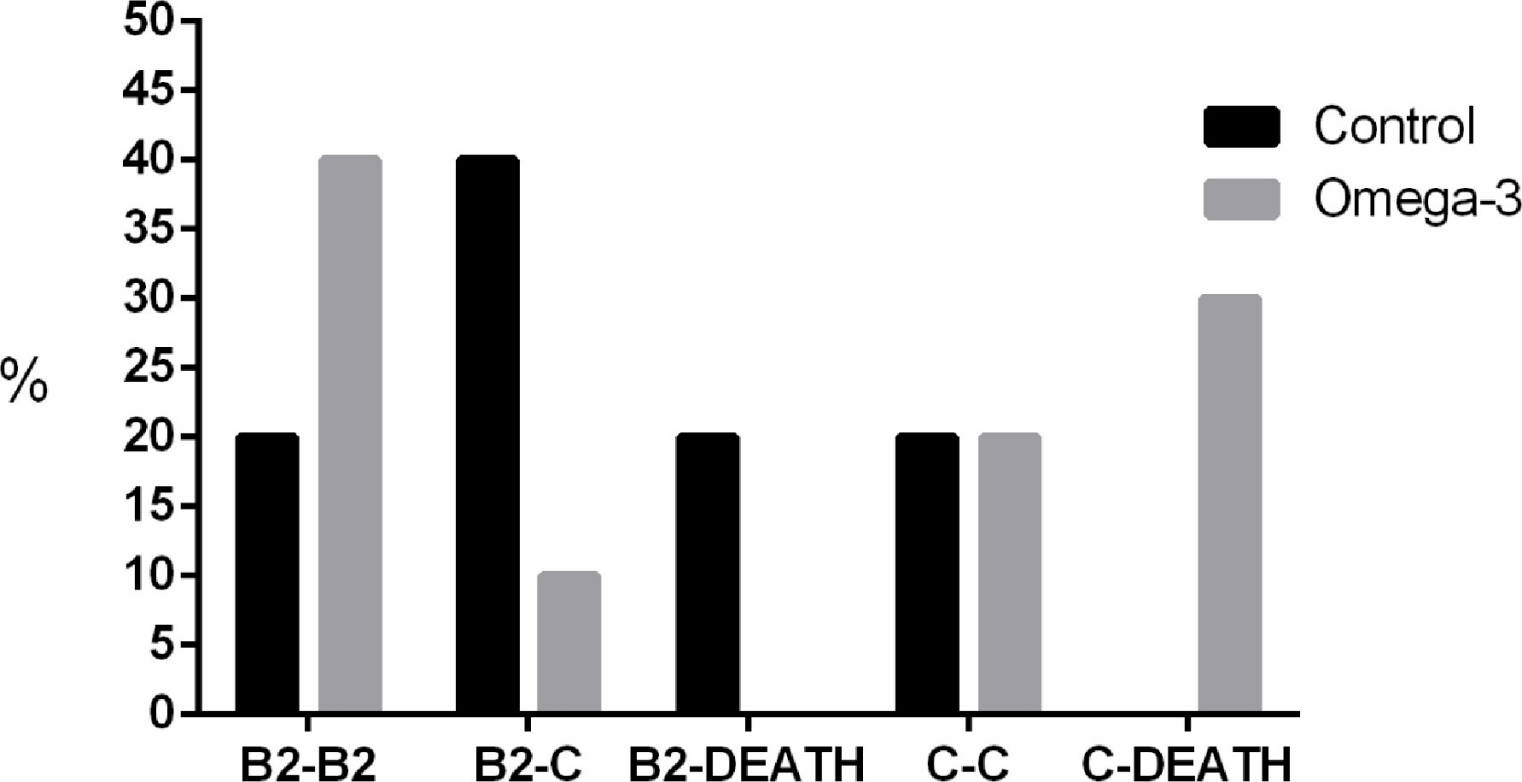
Evolution of disease progression according to the classification proposed by Atkins et al. [4] of control dogs and those submitted to dietary supplementation with omega-3 over a period of 12 months.

Fig 2 illustrates the evolution of MMVD stages over time. The control group presented a higher percentage of animals that evolved from stage B2 to stage C (40%) and from stage B2 to death (20%). The ω-3G presented a higher percentage of evolution between stage C and death (30%) and a higher percentage of dogs remaining in staging B2, with no evolution (40%).

### 3.1. Serum concentrations of inflammatory markers

Concentrations of inflammatory markers are described in Table 3. Regarding IL-1β, differences were found when analyzing treatment by time interaction in control group. For the other cytokines, no differences were found. There was no correlation between BCS, MMS and inflammatory markers concentrations (BCS and IL-1β, p = 0.735; BCS and IL-6, p = 0.108; BCS and TNF-α, p = 0.285; BCS and CRP, p = 0.882; MMS and IL-1β, p = 0.423; MMS and IL-6, p = 0.836; MMS and TNF-α, p = 0.689; MMS and CRP, p = 0.713).

**Table 3:** Serum concentrations of inflammatory markers in dogs with myxomatous mitral valve disease submitted to dietary supplementation with omega-3 for 9 months.

Variables	Times	Treatments				p*	p**
		n	Control	n	Omega-3		
IL-1β (ng/mL)	T0	13	26.75 ± 23.44	16	63.05 ± 77.22	0.143	0.028
	T3	10	31.66 ± 26.76	10	48.68 ± 38.25	0.105	
	T9	9	81.96 ± 68.51	8	105.88 ± 52.23	0.328	
	p***		0.013		0.110		
IL-6 (pg/mL)	T0	13	9.19 ± 7.89	16	23.30 ± 54.47	0.631	0.402
	T3	10	9.56 ± 11.22	10	46.98 ± 126.20	0.684	
	T9	9	297.01 ± 816.51	8	6.23 ± 2.22	0.529	
	p***		0.927		0.726		
TNF-α (pg/mL)	T0	13	9.38 ± 6.68	16	30.99 ± 77.06	0.579	0.410
	T3	10	10.09 ± 9.49	10	51.65 ± 142.49	0.684	
	T9	9	196.23± 506.29	8	5.13 ± 1.09	0.776	
	p***		0.872		0.940		
PCR (ng/mL)	T0	13	36.78 ± 11.72	16	28.70 ± 9.21	0.075	0.311
	T3	10	28.24 ± 15.32	10	40.00 ± 22.93	0.353	
	T9	9	42.65 ± 16.85	8	35.83 ± 20.94	0.181	
	p***		0.105		0.701		

p*: Difference between treatments; p**: Difference between times; p***: Difference in treatment by time interaction; Kruskal-Wallis test at 5% of significance.

### 3.2. Holter analysis

Table 4 describes the number of each type of arrythmia in the experimental groups. CG presented a higher number of arrythmias compared to ω-3G, except for the ventricular premature complexes (VPC) and ventricular tachycardia (VT).

**Table 4:** Number of arrythmias detected in 24 hours of Holter evaluation of dogs with B2 or C stages of myxomatous mitral valve disease supplemented or not with omega-3 for 12 months.

Arrythmias	Time	Treatments									
		Control					Omega-3				
		Max	Md	Q1	Q3	P95	Max	Md	Q1	Q3	P95
Isolated APC	T0	13604	9	0	1552	13604	4910	2	0	22	4910
	T3	9652	4	0	55	9652	400	0	0	36	400
	T6	6315	51	3	115	6315	526	1	0	340	526
	T9	6680	18	0	59	6680	1458	5	0	557	1458
	T12	55	8	0	35	55	37	7	5	12	37
Paired PAC	T0	634	0	0	4	634	164	0	0	1	164
	T3	456	0	0	1	456	0	0	0	0	0
	T6	2784	0	0	1	2784	6	0	0	2	6
	T9	2669	0	0	0	2669	20	0	0	7	20
	T12	5	0	0	1	5	8	0	0	3	8
Isolated PVC	T0	126	2	0	8	126	208	0	0	1	208
	T3	89	3	1	4	89	18	1	0	4	18
	T6	2327	1	0	6	2327	26	1	0	1	26
	T9	546	2	1	12	546	467	0	0	0	467
	T12	79	2	0	3	79	44	0	0	0	44
Bigeminal PVC	T0	1	0	0	0	1	3	0	0	0	3
	T3	2	0	0	1	2	6	0	0	0	6
	T6	4	0	0	0	4	0	0	0	0	0
	T9	2	0	0	0	2	0	0	0	0	0
	T12	2	0	0	0	2	0	0	0	0	0
Atrial tachycardia	T0	135	0	0	1	135	26	0	0	0	26
	T3	2324	0	0	0	2324	0	0	0	0	0
	T6	2575	0	0	0	2575	1	0	0	0	1
	T9	2064	0	0	1	2064	2	0	0	0	2
	T12	1	0	0	1	1	2	0	0	1	2
Ventricular tachycardia	T0	4	0	0	0	4	1	0	0	0	1
	T3	2	0	0	0	2	0	0	0	0	0
	T6	8	0	0	0	8	0	0	0	0	0
	T9	1	0	0	0	1	1	0	0	0	1
	T12	0	0	0	0	0	0	0	0	0	0

PAC: Premature atrial contraction; PVC: Premature ventricular complex; Max.: Maximum; Md.: Median; Q1: 25% of dogs; Q3: 75% of dogs; P95: 95% of dogs.

Control group had 2.96 more chances to present some type of arrythmia compared to the ω-3G group according to the odds ratio analysis (p-value = 0.003; CI = 1.450–6.603).

### 3.3. Doppler echocardiography and arterial blood pressure

Table 5 describes the values for doppler measurements and arterial blood pressure. The 2D mode showed degeneration and thickening of the valvular leaflets with regurgitation in the mitral or tricuspid valves or both of all dogs included in the study. For the 2D and M-mode variables, there was differences in treatment by time interaction for the Ao measures (p = 0.015) with inferior averages in the ω-3G and differences between treatments at T0 for systolic IVS (p = 0.015) and systolic left ventricular free wall thickness (LVFW) (p = 0.043) with lower averages in the ω-3G. When the pulsed wave doppler was performed, differences between treatments at T6 was found only for the maximal systolic pulmonary velocity, with higher averages in the ω-3 group (p = 0.012). Among the averages obtained by tissue doppler, the E’m/A’m ratio showed differences between treatments at T12 (p = 0.042), with lower values in ω-3G.

**Table 5:** Arterial blood pressure and doppler echocardiography measurements of dogs with B2 or C stages of myxomatous mitral valve disease supplemented or not with omega-3 for 12 months.

Variables	Time	Treatments				p*	p**
		n	Control	n	Omega-3		
ABP (mmHg)	T0	13	137.64 ± 18.72	16	128.89 ± 20.52	0.316	0.800
	T3	10	140.47 ± 20.49	10	127.87 ± 26.62	0.393	
	T6	9	142.03 ± 20.38	8	132.15 ± 15.72	0.370	
	T9	9	138.53 ± 20.00	8	130.87 ± 27.05	0.541	
	T12	7	138.28 ± 19.54	7	139.07 ± 19.53	1.000	
	p***		0.993		0.911		
LA (mm)	T0	13	29.44 ± 5.89	16	26.70 ± 5.84	0.316	0.900
	T3	10	24.88 ± 7.53	10	25.11 ± 6.91	1.000	
	T6	9	21.97 ± 7.93	8	22.37 ± 7.80	1.000	
	T9	9	20.32 ± 8.73	8	23.18 ± 7.52	0.541	
	T12	7	20.41 ± 7.25	7	24.26 ± 6.52	0.423	
	p***		0.060		0.479		
Ao (mm)	T0	13	13.47 ± 2.03	16	12.34 ± 1.88	0.201	0.015
	T3	10	17.14 ± 8.53	10	11.93 ± 2.27	0.280	
	T6	9	21.06 ± 10.32	8	13.85 ± 5.94	0.200	
	T9	9	19.91 ± 10.31	8	13.21 ± 5.17	0.236	
	T12	7	19.34 ± 10.29	7	15.86 ± 5.71	1.000	
	p***		0.728		0.659		
LA/Ao	T0	13	2.22 ± 0.49	16	2.20 ± 0.43	0.821	0.820
	T3	10	2.20 ± 0.44	10	2.12 ± 0.55	0.579	
	T6	9	2.15 ± 0.55	8	1.98 ± 0.32	0.815	
	T9	9	2.41 ± 0.49	8	2.12 ± 0.42	0.236	
	T12	7	2.23 ± 0.47	7	2.00 ± 0.32	0.530	
	p***		0.762		0.753		
IVSs (mm)	T0	13	9.91 ± 1.55	16	8.45 ± 1.35	0.015	0.122
	T3	10	9.38 ± 1.57	10	8.14 ± 1.31	0.143	
	T6	9	8.58 ± 2.05	8	7.73 ± 1.40	0.277	
	T9	9	9.04 ± 1.97	8	8.25 ± 1.63	0.321	
	T12	7	8.61 ± 2.00	7	9.20 ± 1.66	0.639	
	p***		0.038		0.428		
IVSd (mm)	T0	13	6.03 ± 1.38	16	5.38 ± 1.00	0.217	0.220
	T3	10	5.47 ± 0.96	10	5.00 ± 0.79	0.243	
	T6	9	5.29 ± 1.15	8	5.21 ± 0.82	0.536	
	T9	9	5.29 ± 1.23	8	5.33 ± 0.90	0.918	
	T12	7	5.65 ± 0.86	7	6.53 ± 0.43	0.073	
	p***		0.788		0.058		
LVFWs (mm)	T0	13	10.21 ± 1.09	16	9.09 ± 1.34	0.043	0.273
	T3	10	10.08 ± 1.39	10	9.13 ± 1.70	0.113	
	T6	9	9.55 ± 1.31	8	9.16 ± 1.18	0.606	
	T9	9	9.89 ± 1.80	8	9.96 ± 1.93	0.837	
	T12	7	10.47 ± 1.96	7	10.77 ± 1.41	0.755	
	p***		0.770		0.254		
LVFWd (mm)	T0	13	5.98 ± 0.97	16	5.39 ± 0.96	0.128	0.749
	T3	10	5.97 ± 1.17	10	5.21 ± 0.97	1.156	
	T6	9	5.65 ± 1.09	8	5.70 ± 1.17	0.918	
	T9	9	5.76 ± 1.38	8	5.68 ± 1.12	0.758	
	T12	7	5.84 ± 0.96	7	6.24 ± 1.01	0.268	
	p***		0.922		0.297		
LVDN (mm)	T0	13	1.97 ± 0.41	16	1.95 ± 0.35	0.964	0.219
	T3	10	2.08 ± 0.45	10	1.90 ± 0.41	0.353	
	T6	9	2.03 ± 0.37	8	1.67 ± 0.34	0.074	
	T9	9	2.04 ± 0.46	8	1.62 ± 0.54	0.200	
	T12	7	1.82 ± 0.41	7	1.66 ± 0.24	0.432	
	p***		0.833		0.381		
EF (%)	T0	13	79.36 ± 6.48	16	78.88 ± 6.99	0.981	0.679
	T3	10	79.00 ± 6.30	10	78.33 ± 6.94	0.720	
	T6	9	76.11 ± 10.10	8	77.36 ± 5.72	0.918	
	T9	9	73.52 ± 14.73	8	79.00 ± 8.89	0.470	
	T12	7	71.38 ± 17.08	7	72.07 ± 17.60	1.000	
	p***		0.643		0.958		
SF (%)	T0	13	47.15 ± 6.34		46.83 ± 6.89	0.867	0.993
	T3	10	47.10 ± 6.33		46.41 ± 6.69	0.780	
	T6	9	44.37 ± 8.33		45.10 ± 5.64	0.918	
	T9	9	45.19 ± 8.02		47.05 ± 7.76	0.536	
	T12	7	44.43 ± 10.20		44.93 ± 11.52	0.876	
	p***		0.720		0.948		

ABP: Arterial blood pressure; LA: Left atrium; Ao: Aorta; LA/Ao: Left atrium aorta ratio; IVSs: Interventricular septum in systole; IVSd: Interventricular septum in diastole; LVFWs: Left ventricular free wall thickness in systole; LVFWd: Left ventricular free wall thickness in diastole; LVDN: Normalized left ventricular diameter; EF: Ejection fraction; SF: Shortening fraction; p*: Differences within each time; p**: Differences in time and treatment interaction; p***: Difference between times within the treatments; Kruskal-Wallis test at 5% of significance.

No differences were found for ABP between the experimental groups and all values were within normality range for dogs [29].

### 3.4. Thoracic radiography

**Fig 3:**
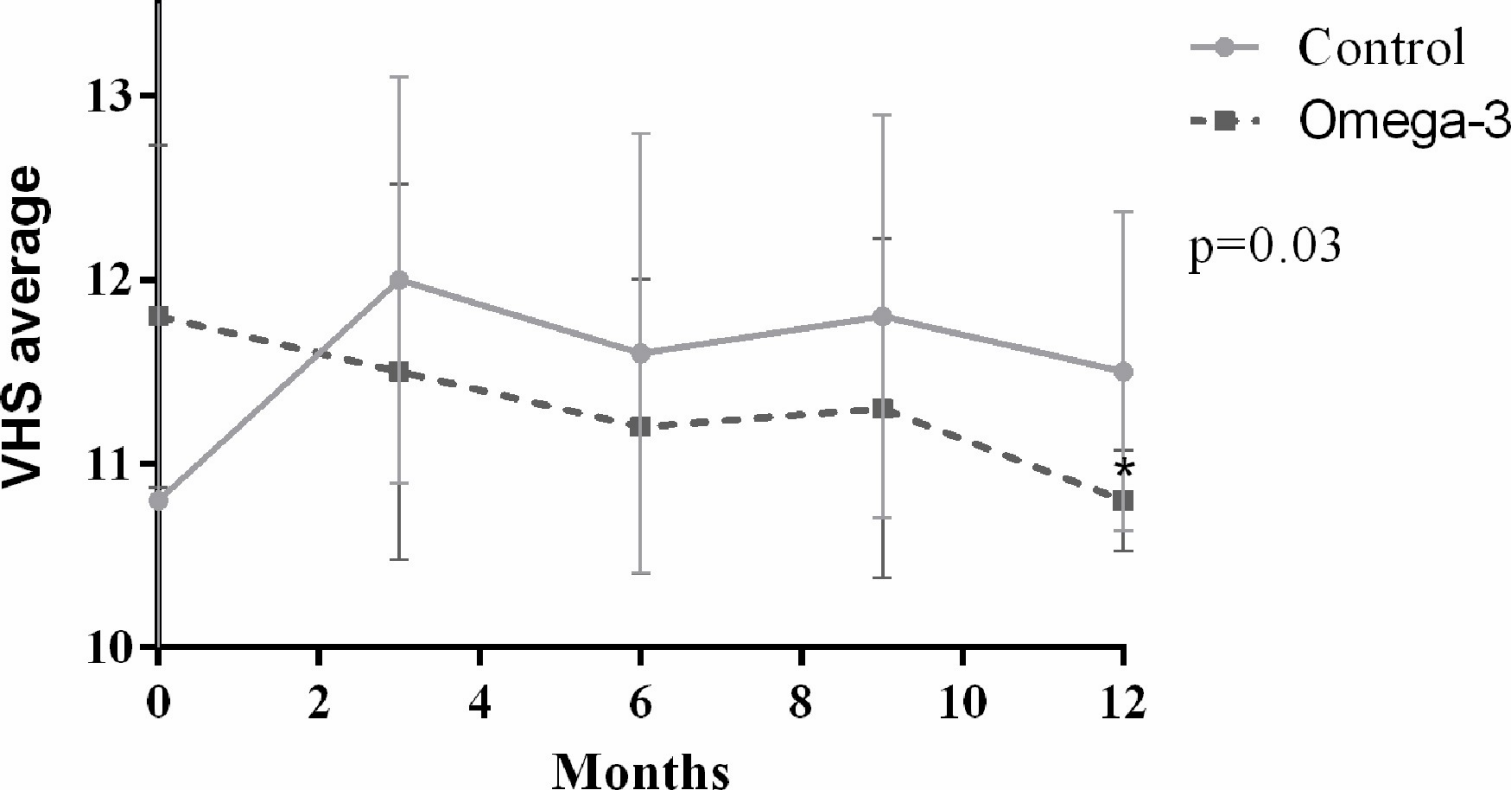
Averages of vertebral heart size (VHS) of dogs with B2 or C myxomatous mitral valve disease with omega-3 dietary supplementation or not for 12 months. *Difference between T0 and T12; Kruskal-Wallis test at 5% of significance.

The results obtained for VHS are shown in Fig 3. No differences were observed between treatments. However, within the ω-3 group, the average of T12 was lower than that of T0. However, it was observed that the means of the CG of stage C dogs increased over time, on the contrary to what occurred in the ω-3 group.

### 3.5. NT-proBNP analysis

**Fig 4:**
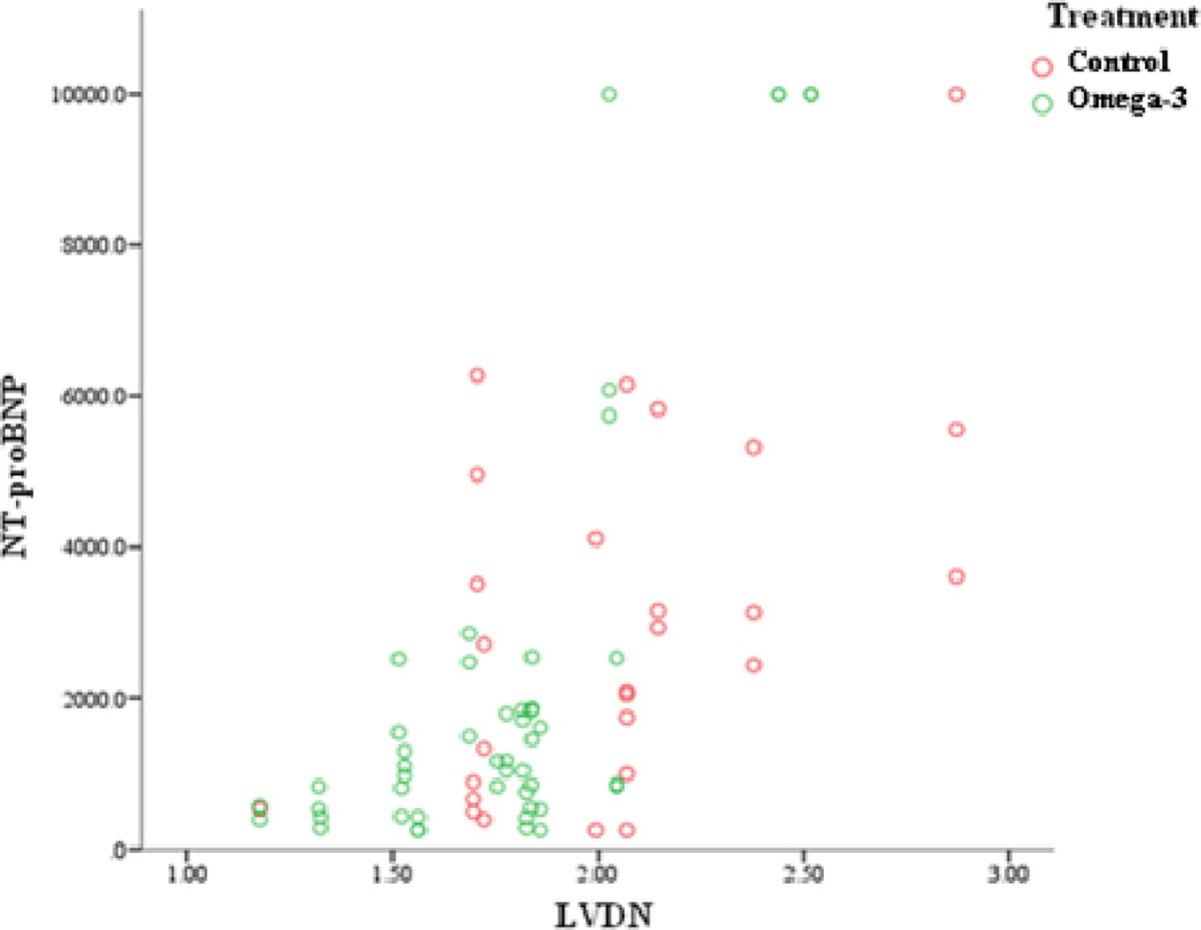
Normalized left ventricular diameter (LVDN) and NT-proBNP correlation.

No differences were found for this marker between the groups (p>0.5) and its values of in treatment by time interaction. The average values for this marker were, respectively for T0, T3 and T6, 530.5±75.4, 456.7±127.2 and 534.2±10.4 for CG and 542.0±61.2, 446.7±146.1 and 525.9±69.5. Although, a positive correlation (p<0.001) between the normalized left ventricular diameter measurements and NT-proBNP averages was identified in both groups. Thus, the higher the normalized LVD, the higher the concentration of NT-proBNP (Fig 4).

### 3.6. Survival

**Fig 5:**
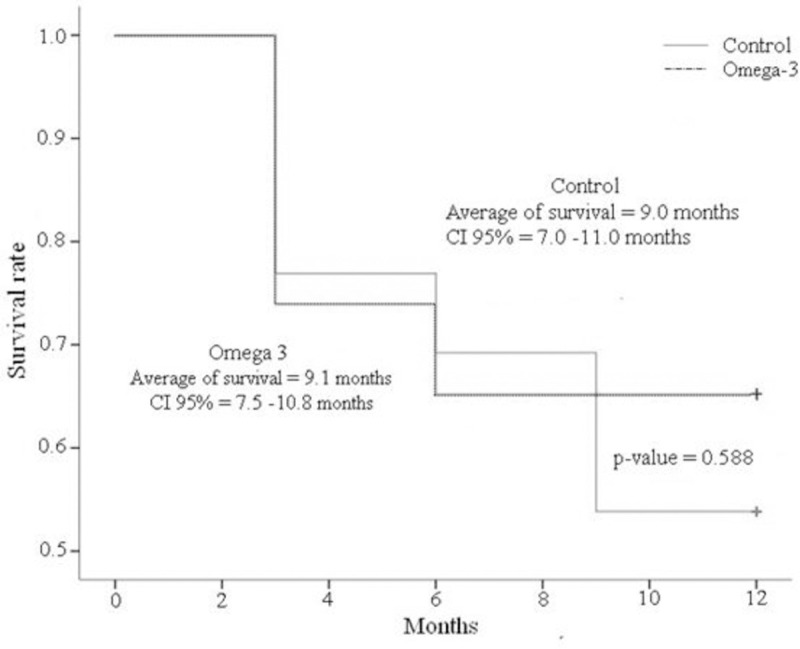
Survival time of dogs with stage B2 and C MMVD with omega-3 dietary supplementation or not (Control) for 12 months.

No differences were found between the treatments on survival (p = 0.588) (Fig 5).

## 4. Discussion

Regarding clinical evaluation, increased RR was observed considering the range of 18-36rpm for dogs, although it is noteworthy that this interval was set for dogs at rest and at a stress-free environment, so under small stressful conditions, tachypnea may occur [31].

All dogs presented different gradings of heart murmur as expected duo to the inclusion criteria, although it was possible to notice that the dogs of the control group evolved more over time and, consequently, a higher percentage developed a more severe form of the disease. However, it was not reflected in the overall survival time when compared to the ω-3 group. Moreover, all dogs received the treatment protocol recommended by Keene et al. [2] and the prescription of any of the medications, specially furosemide, was not influenced by the diet.

The evaluation of BCS and MMS is important in heart disease patients, since the presence of congestive heart failure (CHF) associated to MMVD changes of inflammatory cytokines (TNF-α, IL-1, IL-6, IL-8, chemokines, catecholamines, cortisol and others) induce an inflammatory state and decreases the body’s ability to make metabolic adaptations [18] and use amino acids as a source of energy, causing muscle catabolism that results in the loss of lean mass and the development of cardiac cachexia [4].

No differences were found for these parameters in the groups and between treatment by time interaction. However, the subjective nature of this evaluation may not precisely demonstrate differences of body composition and a more accurate method like dual- X-ray absorptiometry (DEXA) or deuterium isotopes may provide a better comprehension [32].

In the CG, a gradual increase in IL-1β, IL-6, TNF-α and CRP levels was observed, as well as for IL-1β and CRP levels in the ω-3 group. IL-1 induces systemic inflammation by activating the cyclooxygenase-2 metabolic pathway, inducing the production and release of CRP, TNF-α and IL-6 [33]. The results presented here show a gradual increase in IL-1β and CRP levels even in the ω-3G, confirming the presence of an inflammatory effect induced by heart disease and CHF as reported by previous studies [34–36].

IL-6 and TNF-α concentrations increased in the control group and decreased in the ω-3 group. The increase in IL-6 levels is directly correlated to the severity of various diseases, regardless of their etiology [37] and the ω-3 group presented decreasing values of this interleukin over time.

Dogs that died during the study showed clinical signs of cardiac insufficiency before death and the evaluation of the progression of MMVD within the groups showed higher percentage of control dogs that evolved from asymptomatic to CHF and death. In contrast, in the ω-3 group most of them remained asymptomatic throughout the evaluation period, and those who died had already developed CHF when they were included in the study. Patients included in stage C have had at least one episode of CHF and were, therefore, in a more advanced stage of the disease [1].

The ABP averages obtained with the vascular doppler method include normal values for dogs [27]. In human patients supplemented with ω-3, a small dose-dependent hypotensive effect was observed mainly in hypertensive subjects and those with clinical atherosclerotic disease or hypercholesterolemia [15]. This effect was not observed in our dogs possibly because they had their ABP controlled with medications and did not had hypercholesterolemia.

A reduction of systolic ABP was recently observer in a 6-month study in dogs with stage B2 and C of mitral valve disease fed a diet supplemented with a blend of nutrients including medium-chain triglycerides, EPA and DHA, magnesium, taurine and vitamin E [38]. Since omega-3 was included in a similar percentage as in our study, the blood pressure effect was possibly achieved due to the interaction of key nutrients that possibly acted synergistically to achieve the documented efficacy. Therefore, other dietary factors may play an important role like sodium concentration which in our study was lower (43.25mg/100kcal) than in Li et al. study (61.46 mg/kg) [38] and may have contributed to a better control of ABP. All HR averages found in the ambulatory electrocardiogram were within the reference values (60 and 160bpm) for dogs [24] and the averages of the other parameters were also within normal ranges, except for the width (duration) of the P waves, that were greater than 40ms suggesting overload of the left atrium, which is also expected in dogs with MMVD that have atrial enlargement [39].

MMVD may induce a compensatory activation of the sympathetic nervous system (SNS), which leads to increased HR and sinus tachycardia which is also related to the severity of the disease and may be a marker of mortality [40]. Atrial fibrillation is related to an increase in atrial chambers, which may predispose to the onset of premature atrial ectopic beat (APC), atrial tachycardia and atrial fibrillation [41]. In a study with dogs with MMVD, 36% had atrial fibrillation due to valvular degeneration [42].

The predominant rhythm in the ω-3G dogs was the respiratory sinus arrhythmia. This rhythm occurs through vagal mediation and is characterized by HR variation during expiration and inspiration [43]. In the ω-3 group, a higher presence of sinus arrest and atrioventricular block was observed. Both arrhythmias are commonly associated with moments of bradycardia [44]. It is noteworthy that this group also observed a higher prevalence of rhythms related to parasympathetic nervous system mediation, characteristics that are associated with ω-3 fatty acid supplementation [45,46].

Supplementation with ω-3 reduced arrhythmic episodes. Thus, it can be suggested that dogs included in the group treated with ω-3 had lower chances of death due to arrhythmias [47], representing an important benefit to dogs with the disease.

In general, the echocardiographic measurements found in this trial are in line with those expected in dogs in stages B2 and C of MMVD according to the guidelines for the classification of this disease, with emphasis on ventricular remodeling and left atrial dilation [2] and no differences were observed between the experimental groups.

All echocardiograms were performed by the same observer, so it is likely that some variations in the mean values, without statistical significance, were due, in part, to the intra-observer variation as well as to the death of some dogs from the study, over time of analysis.

Dogs presented at all times and in both treatments increased VHS averages (>10.5vertebrae) [30]. However, in the ω-3 group there was a significant reduction throughout the study. The increase in VHS values of dogs with MMVD occurs six to 12 months before the onset of CHF, being greater at the last measure before the episode of congestion [48]. Therefore, for dogs in the ω-3G, reduced VHS values may correspond to a lower volumetric overload, as well as a lower risk of developing CHF. Although there were no differences when compared to the control group and the author of this study attribute this result possibly due to the length of the study. Evaluations of this parameter over a longer period could possibly show differences.

NT-proBNP serum concentrations were equal between groups. The range of values were higher in control dogs, especially those in stage C. The normalized LVD measurement correlated with the means found in NT-proBNP. The mean of the stage B2 dogs were similar to those found in dogs with DVM, as well as the stage C dogs, with those obtained in dogs with CHF [49–52].

BNP is one of the main natriuretic hormones produced and released by the cardiac muscle in response to stimuli such as volume overload, hypertrophy and hypoxia and therefore can be used to verify the degree of cardiac dysfunction [53]. Thus, the greater the ventricular remodelling, the greater will be NT-proBNP serum concentrations, as observed in the present study. It can also be used as a predictor of heart disease and in identifying dogs with CHF [51].

Three dogs that had the highest concentrations (<10000pmol/L) of NT-proBNP died after six months of study initiation. Levels of this biomarker were associated with patient survival [53]. Thus, dogs below 965pmol/L are more likely to survive.

## 5. Conclusions

Dietary supplementation with ω-3 fatty acids contributes to more favourably Doppler echocardiographic measurements, such as normalized LVD, and reduces the VHS measures. NT-proBNP dosages, associated with normalized LVD averages suggest a lower volumetric overload in the ω-3G dogs. Thus, showing a contribution to the control of volumetric overload and, consequently, CHF.

The results found in the electrocardiographic and Holter evaluations confirm the antiarrhythmic effect of ω-3 supplementation reducing by 2.96 the chances of arrhythmias in ω-3G group compared to CG and therefore the cardioprotective effect of EPA and DHA supplementation.

The pathways by which EPA and DHA exert the mentioned cardioprotective effect is still to be better understood. Lionetti et al. [54] and Tremblay et al. [55] have mentioned that mechanisms underlying the regulation of DNA methylation might play a role. In this context, fish oil is capable of enhancing methylation of ATF1 (activating transcription factor 1), which induces the production of atheroprotective macrophages and HDAC4 (histone deacetylase 4), which encodes for muscle differentiation and neuronal survival and, finally, fish oil is capable of reducing methylation of IGFBP5 (insulin-like growth factor binding protein 5), which encodes for the production of this hormone whose increase is observed in young patients with coronary heart disease.
